# The ethical and legal aspects of palliative sedation in severely brain-injured patients: a French perspective

**DOI:** 10.1186/1747-5341-6-4

**Published:** 2011-02-08

**Authors:** Antoine Baumann, Frédérique Claudot, Gérard Audibert, Paul-Michel Mertes, Louis Puybasset

**Affiliations:** 1 Département d'Anesthésie Réanimation, Hôpital Central, Centre Hospitalier Universitaire de Nancy, 29 avenue du Maréchal de Lattre de Tassigny, C.O. n°34, 54035 Nancy Cedex, France; 2 Service de Médecine légale et de Droit de la Santé, Faculté de médecine de Nancy, 9 avenue de la Forêt de Haye, 54505 Vandoeuvre-les-Nancy Cedex, France; 3 Département d'Anesthésie Réanimation, Hôpital Central, C.H.U. de Nancy, Unité INSERM 961, Faculté de médecine de Nancy. France; 4 Service de Neuroréanimation, Centre Hospitalier Universitaire Pitié-Salpêtrière, Assistance Publique-Hôpitaux de Paris, 47-83 boulevard de l'Hôpital, 75651 Paris Cedex 13, France

## Abstract

To fulfill their crucial duty of relieving suffering in their patients, physicians may have to administer palliative sedation when they implement treatment-limitation decisions such as the withdrawal of life-supporting interventions in patients with poor prognosis chronic severe brain injury. The issue of palliative sedation deserves particular attention in adults with serious brain injuries and in neonates with severe and irreversible brain lesions, who are unable to express pain or to state their wishes. In France, treatment limitation decisions for these patients are left to the physicians. Treatment-limitation decisions are made collegially, based on the presence of irreversible brain lesions responsible for chronic severe disorders of consciousness. Before these decisions are implemented, they are communicated to the relatives. Because the presence and severity of pain cannot be assessed in these patients, palliative analgesia and/or sedation should be administered. However, palliative sedation is a complex strategy that requires safeguards to prevent a drift toward hastening death or performing covert euthanasia. In addition to the law on patients' rights at the end of life passed in France on April 22, 2005, a recent revision of Article 37 of the French code of medical ethics both acknowledges that treatment-limitation decisions and palliative sedation may be required in patients with severe brain injuries and provides legal and ethical safeguards against a shift towards euthanasia. This legislation may hold value as a model for other countries where euthanasia is illegal and for countries such as Belgium and Netherlands where euthanasia is legal but not allowed in patients incapable of asking for euthanasia but in whom a treatment limitation decision has been made.

## Introduction: why use palliative sedation?

Every year, millions of people with serious diseases, as well as their loved ones, are confronted with decisions relating to the quality of the time that remains to be lived. The quality of the end of life may be severely altered by pain or other distressing symptoms. Physicians have a duty to relieve suffering in their patients. However, in most countries, they are not allowed to intentionally shorten life, although treatments given to relieve suffering are permitted even if they are also expected to shorten life (double-effect principle). A major challenge faced by physicians is to honour their patients' wishes and values and to help them safeguard their dignity and peace at the end of life.

Some seriously ill patients, such as those with severe brain injuries, are unable to communicate their suffering. In these patients, the effectiveness of palliative care cannot be assessed: the possibility of persistent suffering cannot be ruled out [1]. There is a wide consensus that palliative sedation is appropriate as a last resort in this situation [2]. However, palliative sedation is a complex intervention that is closer to physician-assisted suicide and voluntary active euthanasia than is ordinarily acknowledged. Safeguards are needed whenever a medical intervention may hasten death. Legislation stating which practices are permissible would reassure the numerous patients who fear a "bad" death and would improve practice uniformity among physicians [3]. The French law on patients' rights and the end of life passed on April 22, 2005 (Law n° 2005-370, known as the Leonetti law) [4] indicates that patients should be allowed to die as comfortably and peacefully as possible but should not be made to die. This law reflects the evolution of French medical thinking about the best means of protecting patients' well-being and dignity while refraining from intentionally causing death. It has also led to further ethical discussions, most notably about palliative sedation after treatment-limitation decisions in patients with severe brain injuries. Thus, a committee evaluated the Leonetti law and revised the article of the French Code of Medical Ethics relating to the relief of suffering [5]. These issues were specifically addressed during multiple hearings in the French Parliament [6]

## Specific issues raised by suffering in patients with severe brain injuries

Severely brain-injured patients who are unable to communicate are at high risk for suffering. More specifically, the ability to perceive pain has been well documented in patients who are in a minimally conscious state [1,7,8], in whom suffering is very difficult to assess [9]. In these patients, when treatment-limitation decisions are made based on the clinical findings and results of investigations, the potential appropriateness of palliative sedation is an extremely relevant issue [10]. The possible need for palliative sedation deserves special attention when patients are taken off mechanical ventilation and either extubated or decannulated, as well as when nutrition and hydration are stopped in patients with chronic consciousness disorders [11]. Currently available scientific knowledge usually does not allow the distinction between unconscious nervous reactions and pain perception in most such patients [1], and the possibility of "unconscious pain" has been raised [12], generating considerable interest in the issue of palliative sedation. The goal of palliative sedation started before or at the time life-supporting treatments are withdrawn is to eliminate pain perception and neurological responses that might result from treatment withdrawal. In this situation, palliative sedation is a gesture of humanity towards both the patient and the family members, whose primary request is that their loved one does not suffer. Palliative sedation can thus represent an appropriate form of palliative care.

## The nature of palliative sedation and the differences with euthanasia and physician-assisted suicide

The concept of sedation does not have a precise medical content. Literally, "to sedate" means to alleviate suffering. The definition of palliative sedation remains vividly debated, particularly regarding the presence or absence of an intention to hasten death [13]. Palliative sedation is defined by some authors as the use of drugs to put the patient in a state of comfort and unawareness of his or her situation without intentionally hastening death [14]. The level of palliative sedation varies with the drugs used, and the above-mentioned definition covers the continuum from simply keeping the patient asleep to inducing an artificial coma. Inducing an artificial coma, which can be described as major sedation, may be indispensable in the event of treatment limitations such as the withdrawal of life-supporting interventions. Major palliative sedation has complex links with treatment withdrawal, as underlined in the parliamentary report on the evaluation of the Leonetti law (p. 204-216) [15]. (Rapport de la mission d'évaluation de la loi n° 2005-370 du 22 avril 2005). Palliative sedation has been criticized as being a slow, disguised, and socially acceptable form of euthanasia [16-21]. Other authors endorse the "double effect" principle [14,22,23], acknowledging that a shorter time to death is a possible side effect of palliative care [24,25].

The main difference between physician-assisted suicide or euthanasia and palliative sedation lies in the presence or absence of an intention to hasten death and the precise knowledge of the patient's wishes [23]. The strict definition of euthanasia retained by the Dutch and Belgian laws is "the intentional taking of someone's life by another, at his request". In palliative sedation, the drug dosages are increased only until the suffering is alleviated. The intention in palliative sedation is to fully protect the patient from pain. There is no intention to hasten death. In addition, palliative sedation is reversible, whereas death caused by an overdose of sedatives is not [26]. Allowing a patient to die at some point may constitute a practical necessity if medical care is to be given successfully, but the same is not true of physician-assisted suicide [27]. Moreover, support for palliative sedation is widespread among internists. Most physicians who view palliative sedation favourably do not support physician-assisted suicide and feel that these two practices are on different sides of the line separating the ethical from the unethical [28].

## The French context

The issue of end-of-life care was long neglected by French law. Healthcare providers could rely only on the French code of medical ethics, which required that they "refrain from any unreasonable obstinacy in investigations or treatments" while reminding them solemnly that they had "no right to cause death intentionally" (Article 38, Additional file 1). This last provision was the translation to the medical ethics field of Penal Code laws prohibiting homicide. The French Code of Medical Ethics is part of the Public Health Code and has the force of law.

In addition to requiring that healthcare providers respect patients' decisions to stop treatment, the Leonetti law allows physicians to make treatment withdrawal decisions for patients who are "unable to express their wishes" (Articles L. 1111-4 and L. 1111-13), as a means of avoiding unreasonable obstinacy, provided the decisions are preceded by consultation of all medical team members, any advance directives written by the patient, and the close relatives, and are considered appropriate by an external consultant [4]. French intensive care societies view tube feeding as a life-sustaining treatment whose withdrawal is advisable when all treatments except comfort care are stopped.

When a decision is made to withdraw treatments intended to prolong life, the physician must bear in mind that the law authorizes the withdrawal of such treatments only with the condition that any additional suffering potentially caused by treatment withdrawal will be completely eliminated by palliative care.

## French legislation on palliative sedation

A parliamentary mission known as the Leonetti Mission was appointed by the French government to assess the implementation of the 2005 law. It released its conclusions in late 2008. The Leonetti Mission called into question the adequacy of palliative care in severely brain-injured patients with treatment-limitation decisions taken in a collegial manner. In some of these patients, pain is not readily assessable and the existence of pain cannot be ruled out. In 2006 in France, a young man in a vegetative state whose gastric tube was withdrawn without preliminary sedation died only 6 days later after experiencing multiple seizures that caused severe distress to the family. This case, together with the Leonetti Mission conclusions, promoted a revision of Article 37 of the French code of medical ethics. A paragraph was added to this article to require the use of sedation and/or analgesia to eliminate any suffering possibly caused by treatment withdrawal in patients with brain damage precluding a reliable evaluation of pain perception. The revision of Article 37 was released in January 2010 (Additional file 2). The use of sedation/analgesia under the conditions of transparency and collegiality required by the law will now ensure that patients do not suffer, particularly those in neurointensive care units or neonatology wards. On the legal level, this new requirement confirms the right of patients to palliative care, as stated by article L. 1110-9 of the Public Health Code. It reminds physicians of their duty to respect the patient's right to receive palliative care, including major palliative sedation when considered proportionate to the patient's potential suffering.

Situations involving severe brain injury are not all identical. Current legislation in France is based on the principle of proportionality. Physicians must choose among available treatments those that are proportionate to the patient's condition. Thus, useless treatments must be stopped and palliative strategies selected according to the patient's needs. Among these strategies, deep palliative sedation is allowed by the revised law to eliminate potential suffering in patients whose predicted neurological outcome dictates the withdrawal of life-supporting treatments. The physician must select the level of sedation that is proportionate to the suffering endured, or possibly endured, by each individual patient. In patients with severe brain injuries, the presence and intensity of the pain cannot be reliably assessed and, consequently, the proportionate amount of sedation cannot be determined. The French Board of Physicians has stated that "the goal is to provide patients with severe brain injuries precluding a reliable evaluation of suffering with the same level of relief as that received by patients who are able to communicate." The law requires documentation in the medical files of all the information needed to make the decision. In other words, the decision must be substantiated.

## French law affords legal and ethical safeguards against a shift towards euthanasia

The Leonetti Mission recommended that the Code of Medical Ethics specify the sedation modalities that must accompany the withdrawal of life-supporting treatments in patients whose pain perceptions cannot be assessed. The principle of proportionality of sedation is deemed to protect against the use of sedation with the intention of causing death [29].

The hospital staff may sometimes decide to use sedation because no palliative care team is available to ensure analgesia and to accompany the dying patient. However, deep sedation is in no case a substitute for palliative care, i.e. an easy solution intended to compensate to some extent for the absence of palliative care. Using deep sedation as a substitute for palliative care disregards the principle of proportionality on which the Leonetti law is based.

Transparency of the decision-making process, collegial decision making, information of the relatives, and documentation of the entire decision-making process in the medical files are legal requirements that constitute safeguards against the inappropriate use of palliative sedation. The support and guidance provided by the healthcare team to the family are invaluable in helping the family to accept the last phase of their loved one's life.

## Difficulties, concerns, and reservations

### Technical concerns

- Indication for sedation/analgesia: A crucial challenge is determining which non-communicating brain-injured patients need palliative sedation. Classical clinical criteria for possible pain consist of high blood pressure, tachycardia, tachypnea, wincing, and motor reactions to noxious stimuli [9]. An EEG or functional MRI response to noxious stimuli suggests pain, but the usefulness of these methods for identifying patients who require sedation/analgesia has not been assessed. It seems that only patients in a minimally conscious state can perceive pain and anxiety and therefore usually require analgesics and sedatives [1]. In contrast, patients in a persistent vegetative state can process noxious stimuli only as far as the primary cortex level, which remains disconnected from the level involved in pain sensation, so that no pain is felt [9]. However, the exact nature of the consciousness disorder may be extremely difficult to determine, at least at some points in time [30]. Patients who seem calm and have no hemodynamic or neurological signs of pain probably do not need sedation. Indeed, similar to other medical procedures at the end of life, palliative sedation should meet criteria for prudent practice [31].

- Continuous sedation has several drawbacks and pitfalls. The criteria used to assess the depth and appropriateness of sedation deserve discussion. Sedation can preclude an assessment of the neurological status of the patient by altering the neurological findings from one evaluation to the next. In addition, the accumulation in the body of drugs used to achieve lasting sedation may lead to oversedation. Thus, drugs with short half-lives deserve preference for palliative sedation.

### Ethical concerns

Palliative sedation has been criticized by some authors as a disguised form of euthanasia [17,32]. Others, such as U.S. Supreme Court Justice Sandra Day O'Connor in 1997, have endorsed the practice, arguing that "a patient who is suffering from a terminal illness and who is experiencing great pain has no legal barrier to obtaining medication, from qualified physicians, to alleviate that pain, even to the point of causing unconsciousness and hastening death" [33].

One of the major objections to terminal sedation is that its intention may be to kill the patient in order to alleviate the symptoms, i.e., to seek the double effect instead of just accepting it [34]. The goal of palliative care, by contrast, is to relieve pain, although drugs such as opioids can shorten life. Intent is crucial in law and in ethics, and the double-effect principle states that the foreseeable adverse consequences of treatment are acceptable only if they are unintended [24]. However, a Dutch study has established that many physicians who use palliative sedation do so with the conscious intention of ending their patients' lives, inducing a coma and withholding life-sustaining treatment as a form of "slow euthanasia" [16] that is more acceptable to them and to their patients' families than direct euthanasia [35]. A second objection is that palliative sedation may be used without the patient's consent and that this situation is indistinguishable from involuntary euthanasia [36]. A descriptive study conducted in the Netherlands by Rietjens and colleagues [37] to assess physician behaviour toward patients near the end of life found that patients or their surrogates did not always give their informed consent. Although French law does not require informed consent, the French Board of Physicians has stated in comments on the code of medical ethics that "Transparency is of the highest importance and there should be no doubt in anyone's mind that the decision was pondered carefully and discussed extensively. At all the stages of the procedure, the person of trust or family or, if either is unavailable, close friends must be informed of the issues, steps taken, decisions made, and reasons for those decisions. They must be consulted and heard, and their requests - even if they cannot always be satisfied - must be welcomed and considered." A third objection is that physicians may use palliative sedation even when other ethically preferable interventions are feasible [38]. Pain was the most common reason for palliative sedation in the study by Rietjens and colleagues, although state-of-the-art palliative care can provide satisfactory pain control in 90% of cases [37]. Dutch physicians acknowledge that their intent is to end life and do not uniformly seek less drastic approaches to alleviate suffering [37]. The guidelines on palliative sedation used in many countries require confirmation of the prognosis, consideration of alternative approaches, and collection of informed consent before starting the procedure [39]. Only when these requirements are met consistently will palliative sedation be truly a treatment of last resort.

### Potential interaction with non-heart beating donation and risks of drift

The patients to whom Article 37 applies are potential type III non-heart-beating (NHB) donors according to the Maastricht classification, that is, potential donors awaiting cardiac arrest after withdrawal of life-sustaining treatments in the intensive care unit because of a poor neurological prognosis [40]. Some authors believe that severely brain-injured patients who are dependent on mechanical ventilation but do not meet criteria for brain death are good candidates for NHB organ donation and that terminal sedation is a component of the care given to these NHB donors [41]. French organ donation authorities consider that there can be a conflict of interest between treatment limitation and organ donation and have instituted a moratorium for organ harvesting from Maastricht III patients. However, the issue of organ donation by type III NHB donors is being re-examined in France. Similarly, there may be a conflict of interest between sedation for possible pain and cardiac arrest for Maastricht III organ donation. Thus, palliative sedation might come to be used to hasten death in order to allow Maastricht III organ donation. In some European countries where palliative sedation is widely practiced at the end of the life and Maastricht III organ donation is allowed, physicians use major sedation in combination with the withdrawal of life-sustaining treatments with the intent to hasten death. Thus, there seems to be at best a very tenuous line between palliative sedation and ensuring that death occurs at a time that will benefit a third party. Some authors claim that this issue is not relevant if the patient is a voluntary organ donor at the end of life and that the only important point in this situation is achieving successful organ transplantation [42]. Thus, whether there is a potential conflict of interest when the patient's desire to be an organ donor is documented in advance directives or attested to by a healthcare proxy deserves discussion.

Many other ethical problems arise. More specifically, the patient's desire to be an organ donor may seem to come into conflict with allowing the patient to die with dignity surrounded by his or her loved ones. Furthermore, compliance with the ethical principles underlying palliative care may be difficult in a patient receiving organ preservation procedures in the intensive care unit. In particular, the line between terminal palliative sedation and euthanasia is unclear when mechanical ventilation is removed under deep sedation and neuromuscular-blocking agent administration, as already performed in some hospitals in Belgium. In contrast, palliative sedation as defined by the new French Article 37 must be personalized and tailored to the potential severity of possible pain, with no intent to shorten life. Moreover, it is still extremely difficult to establish with complete certainty that a patient will never recover consciousness.

## Conclusion

In recent years, progress has been made in defining the components of high-quality care for patients with severe consciousness disorders. These components include palliative care to maximize quality of life as an integral part of the overall treatment plan, as well as clarity about the availability of last-resort options when the presence and intensity of pain cannot be assessed.

Indeed, there is a widespread consensus that high-quality palliative care may fail to provide adequate relief. In this situation, good practice requires that palliative care include a consideration of palliative sedation [43]. The existence of brain damage that precludes an assessment of pain produces a similar situation. The French Code of medical ethics specifies that providing analgesia and sedation as needed is a duty when treatment-limitation decisions are implemented in unconscious adults or in neonates. The purpose of recent French legislation is to make palliative sedation available as a last resort to all patients in France, as part of standard care.

Defining palliative sedation for non-communicating or minimally conscious patients is crucial to ensure that a satisfactory balance is achieved between respecting the fundamental right of patients to have their suffering relieved and protecting patients against a shift towards euthanasia.

The end of life should not be synonymous with sedation. Physicians must resist requests for a scheduled death. Palliative sedation should be used only for the right reasons, after a careful decision-making process and in a medically and technically appropriate way [31]. We must bear in mind that palliative sedation can easily shift to slow covert euthanasia [44]. We must continue the time-honoured practice of refraining from deliberately terminating human life [31]. The rule of proportionality of the sedation to the patient's condition and symptoms affords a safeguard against intentionally causing or hastening death.

The recent revision of Article 37 of the French code of medical ethics relating to relief of pain attempts to address all concerns about the possible hazards associated with palliative sedation. In the near future, surveys will be needed to assess compliance with this revised article in everyday practice.

## Competing interests

The authors declare that they have no competing interests.

## Authors' contributions

LP initiated the article and made substantial revisions to the earlier draft. AB did the bibliographic research and wrote the first draft and the final version of the manuscript. FC, GA and PMM read the first version and contributed editorial and critical modifications. All authors read and approved the final manuscript.

## About the author

Antoine Baumann, MD, DESA, is a staff anesthesiologist and intensivist in the Department of Anesthesiology and Surgical Intensive Care of Nancy University Hospital (France) and member of the ethics reflection committee of the hospital. Working in Neurosurgical intensive care unit and middle care unit, he his every day confronted by decision and care in severely brain-injured patients.

## Supplementary Material

Additional File 1 **Article 38 of the French Code of Medical Ethics (Article R.4127-38 of the French Public Health Code)**. Click here for file

Additional File 2 **Revised Article 37 of the French Code of Medical Ethics (Article R. 4127-37 of the French Public Health Code)**. Click here for file
