# Supplementary material for: The ethical and legal aspects of palliative sedation in severely brain-injured patients: a French perspective
Source: Philos Ethics Humanit Med. 2011 Feb 8;6:4. doi: 10.1186/1747-5341-6-4 (PMC3041748; doi:10.1186/1747-5341-6-4)
Supplement: Additional File 1 — Article 38 of the French Code of Medical Ethics (Article R.4127-38 of the French Public Health Code). [file 1747-5341-6-4-S1.DOC]

**Article 38 of the French Code of Medical Ethics (Article R.4127-38 of the French Public Health Code)**

___________________________________________________________________________

“The physician must accompany the dying patient until the very last moment, provide appropriate care and other measures to maximize the quality of the end of life, safeguard the dignity of the patient, and comfort the relatives.

The physician does not have the right to intentionally cause death.”

(Note: this translation was done by the authors and is not official)
