# Supplementary material for: The ethical and legal aspects of palliative sedation in severely brain-injured patients: a French perspective
Source: Philos Ethics Humanit Med. 2011 Feb 8;6:4. doi: 10.1186/1747-5341-6-4 (PMC3041748; doi:10.1186/1747-5341-6-4)
Supplement: Additional File 2 — Revised Article 37 of the French Code of Medical Ethics (Article R. 4127-37 of the French Public Health Code). [file 1747-5341-6-4-S2.DOC]

**Revised Article 37 of the French Code of Medical Ethics (Article R. 4127-37 of the French Code of Public Health)**

___________________________________________________________________________

“I - In all circumstances, the physician must endeavour to alleviate the patient’s suffering by using means appropriate for the patient’s state of health and to provide the patient with moral support. The physician must refrain from unreasonable obstinacy in the use of investigations or treatments and may refrain from starting or continuing treatments that appear pointless or disproportionate or that have no other aim or effect than the artificial maintenance of life.”

“II - In the situations set forth in the fifth paragraph of article L. 1111-4 and the first paragraph of article L. 1111-13, the decision to limit or to stop ongoing treatments must not be made without a previous collegial procedure. The physician may take the initiative to start the collegial procedure. The physician must start the collegial procedure if requested in the patient’s advance directives presented by any of the individuals listed in Article R1111-19 or if requested by the person of trust or family or, if neither is available, a close friend. The individuals entrusted with the patient’s advance directives, the person of trust or family or, if neither is available, a close friend must be informed of the decision to initiate the collegial procedure as soon as it is taken.”

“Treatment withdrawal and withholding decisions should be taken by the physician in charge of the patient, after discussion with the other members of the healthcare team if any exists, and in accordance with the substantiated recommendation of at least one other physician called in as a consultant. There must be no hierarchical ties between the physician in charge of the patient and the consultant. If the physician in charge of the patient and/or the consultant feel a second opinion would be useful then a substantiated recommendation from a second consultant must be obtained.”

“Treatment withdrawal and withholding decisions should take into account the wishes the patient may have expressed previously, in particular in advance directives if available; and the opinion of the person of trust designated by the patient or of the family or, if neither is available, a close friend.”

“When making treatment withdrawal and withholding decisions for a minor or an adult under guardianship, the physician must collect the opinion, depending on the situation, of the individuals holding parental authority or guardian, except when a need for emergency care leaves no time to collect said opinion.”

“The reasons for treatment withdrawal or withholding must be explained. The opinions provided, the nature and content of the discussions among the healthcare team members, and the reasons for the decision must be recorded in the patient’s medical file. The person of trust, if one was designated by the patient or the family or, if neither is available, a close friend must be informed of the nature of, and reasons for treatment withdrawal and/or withholding decisions. Should the physician decide not to comply with the patient’s advance directives, the reasons for this decision must be explained in the patient’s medical file.”

“III - When treatment withdrawal or withholding is decided in accordance with Article L.1110-5 and Articles L1111-4 and L1111-13, under the conditions considered in paragraphs I and II of the present article, and even if the patient has brain damage that precludes an assessment of his or her suffering, the physician must use the treatments, including analgesics and sedatives, designed to accompany the patient in compliance with the principles, and under the conditions, stated in Article R4127-38. The physician must also make sure that the patient’s relatives are informed of the situation and receive the support they need.”

(Note: this translation was done by the authors and is not official)
